# Association between the introduction of a national targeted intervention program and the incidence of surgical site infections in Swiss acute care hospitals

**DOI:** 10.1186/s13756-023-01336-7

**Published:** 2023-11-24

**Authors:** Marcus Eder, Rami Sommerstein, Arlette Szelecsenyi, Alexander Schweiger, Matthias Schlegel, Andrew Atkinson, Stefan P. Kuster, Danielle Vuichard-Gysin, Nicolas Troillet, Andreas F. Widmer, for Swissnoso, Carlo Balmelli, Delphine Berthod, Niccolò Buetti, Stephan Harbarth, Philipp Jent, Jonas Marschall, Hugo Sax, Laurence Senn, Sarah Tschudin Sutter, Aline Wolfensberger, Walter Zingg

**Affiliations:** 1 Swissnoso, the National Center for Infection Control, Bern, Switzerland; 2 Department of Infectious Diseases, Bern University Hospital, University of Bern, Bern, Switzerland; 3 Faculty of Health Sciences and Medicine, University of Lucerne, Lucerne, Switzerland; 4 Department of Medicine and Infectious Diseases, Cantonal Hospital Zug, Zug, Switzerland; 5 Department of Infectious Diseases and Hospital Epidemiology, Cantonal Hospital St Gallen, St Gallen, Switzerland; 6 Division of Infectious Diseases, Washington University in St. Louis School of Medicine, St. Louis, MO USA; 7 Infectious Diseases, Thurgau Hospital Group, Muensterlingen and Frauenfeld, Switzerland; 8 Service of Infectious Diseases, Central Institute, Valais Hospitals, Sion, Switzerland; 9 Department of Infectious Diseases, University Hospital Basel, Basel, Switzerland

**Keywords:** Surgical site infection, Surveillance, Multimodal intervention bundles, Preoperative management, Hair removal, Skin disinfection, Perioperative antimicrobial prophylaxis

## Abstract

**Background:**

In Switzerland, the national surgical site infection (SSI) surveillance program showed a modest decrease in SSI rates for different procedures over the last decade. The study aimed to determine whether a multimodal, targeted intervention program in addition to existing SSI surveillance is associated with decreased SSI rates in the participating hospitals.

**Methods:**

Prospective multicenter pre- and postintervention study conducted in eight Swiss acute care hospitals between 2013 and 2020. All consecutive patients > 18 years undergoing cardiac, colon, or hip/knee replacement surgery were included. The follow-up period was 30 days and one year for implant-related surgery. Patients with at least one follow-up were included. The intervention was to optimize three elements of preoperative management: (i) hair removal; (ii) skin disinfection; and (iii) perioperative antimicrobial prophylaxis. We compared SSI incidence rates (main outcome measure) pre- and postintervention (three years each) adjusted for potential confounders. Poisson generalized linear mixed models fitted to quarter-yearly confirmed SSIs and adjusted for baseline differences between hospitals and procedures. Adherence was routinely monitored through on-site visits.

**Results:**

A total of 10 151 patients were included, with a similar median age pre- and postintervention (69.6 and IQR 60.9, 76.8 years, vs 69.5 and IQR 60.4, 76.8 years, respectively; *P* = 0.55) and similar proportions of females (44.8% vs. 46.1%, respectively; *P* = 0.227). Preintervention, 309 SSIs occurred in 5 489 patients (5.6%), compared to 226 infections in 4 662 cases (4.8%, *P* = 0.09) postintervention. The adjusted incidence rate ratio (aIRR) for overall SSI after intervention implementation was 0.81 (95% CI, 0.68 to 0.96, *P* = 0.02). For cardiac surgery (n = 2 927), the aIRR of SSI was 0.48 (95% CI, 0.32 to 0.72, *P* < 0.001). For hip/knee replacement surgery (n = 4 522), the aIRR was 0.88 (95% CI, 0.52 to 1.48, *P* = 0.63), and for colon surgery (n = 2 702), the aIRR was 0.92 (95% CI, 0.75 to 1.14, *P* = 0.49).

**Conclusions:**

The SSI intervention bundle was associated with a statistically significant decrease in SSI cases. A significant association was observed for cardiac surgery. Adding a specific intervention program can add value compared to routine surveillance only. Further prevention modules might be necessary for colon and orthopedic surgery.

**Supplementary Information:**

The online version contains supplementary material available at 10.1186/s13756-023-01336-7.

## Keypoints

- *Question* Is a targeted surgical site infection (SSI) intervention bundle on top of an existing national surveillance system associated with lower SSI incidence?
- *Findings* In this multicenter intervention study involving 10 151 patients, the introduction of an SSI bundle with three elements to optimize preoperative management (hair removal, skin disinfection, and perioperative antimicrobial prophylaxis) was associated with a significant (19%) decrease in the overall SSI incidence rate ratio. For specific procedure types, the bundle was associated with a significant SSI decrease in cardiac surgery.
- *Meaning* The Swiss surveillance system has been fundamental in characterizing SSI epidemiology and defining priorities for decreasing SSI rates over the past decade. Adding an SSI intervention module at a national level can contribute to lowering SSI incidence and eliminating the preventable proportion of SSIs.

**Supplementary Information:**

The online version contains supplementary material available at 10.1186/s13756-023-01336-7.

## Background

Surgical site infections (SSIs) are among the most frequent types of healthcare-associated infections (HAIs), accounting for approximately 20% of all HAIs [1, 2]. Between 1 and 20% of surgical patients develop SSI [3–5]. Risk factors include the patient’s underlying diseases, type of surgery, and adherence to SSI prevention guidelines. SSIs cause substantial morbidity, mortality, and additional costs and pose a significant burden on healthcare systems [6, 7]. Specifically, SSIs were found to prolong hospital stay by an average of 7–10 days and to increase the risk of death by 2–11 times, at an average cost of between $3000 and $29000 and a doubling of surgical costs [8, 9].

National and international guidelines provide evidence-based measures to reduce SSI risk [6–8, 10–14]. With more than a 50% potential reduction in infection rates, SSIs are estimated to be among the most preventable HAIs [15]. It is debated whether surveillance alone can consistently lower the SSI rate. A large international cohort study including several million operations across multiple SSI surveillance networks detected a steady decline in SSI rates across different procedures over time [16]. In Switzerland, the national SSI surveillance program showed a modest decrease in SSI rates for different types of procedures, including heart, colon, and hip surgery, since its introduction in 2011 [17]. However, the need for structured and mandatory quality improvement efforts was highlighted to achieve a further decrease in SSI rates. [18]

A large multicenter study in the United States demonstrated an intervention bundle associated with a statistically significant decrease in complex *Staphylococcus aureus* SSIs [19]. Similarly, a recent systematic review on nonpathogen-specific bundled interventions in hip arthroplasty showed a significant reduction in SSIs [20]. In the Netherlands, adherence to a surgical care bundle significantly reduced the risk of SSIs relating to different types of surgical procedures [21]. In contrast, Anthony et al. [22] found a 2.5-fold increase in SSI in colorectal patients, warranting a review of the prevention bundle. Given the scarcity of studies using a prevention bundle for different types of surgery, we initiated a study implementing a prevention bundle to compare SSI rates pre- vs postintervention for three different types of surgery.

Aiming to reduce SSI rates in Switzerland, Swissnoso (the National Center for Infection Prevention; www.swissnoso.ch) offered a national, multimodal, targeted intervention program on top of the SSI surveillance for implementation at the hospital level. The aim of this prepost intervention study based on data from the Swiss SSI surveillance program was to determine the effect of a multimodal intervention bundle on SSI rates in the participating hospitals and the potential added benefit of the intervention program compared to surveillance alone.

## Methods

### Study design and data sources

Eight acute care hospitals in Switzerland participated in this multicenter prepost intervention study. Following a national call, the hospitals voluntarily signed up for the study and started implementing the intervention bundle between January 2016 and July 2017. The hospitals continued to provide data as part of the mandatory national SSI surveillance program by prospectively collecting SSI rates for all cardiac, colon, and hip/knee replacement surgeries. All consecutive patients aged > 18 years were included with no exclusion criteria. The follow-up period was 30 days for all surgeries and a 1-year follow-up for implant-related surgeries. All patients with at least one follow-up were included. Initially, nine Swiss acute care hospitals agreed to participate in the intervention, one of which had to be excluded because no data on compliance with the process parameters were collected. The remaining eight participating hospitals included larger and smaller (public and private) institutions across different geographical regions, representing healthcare institutions in all regions of Switzerland. Overall postdischarge follow-up rates were greater than 91% [17, 18]. A description of the Swiss SSI surveillance system is provided under Additional file 1: Supplementary Information, in previous publications, [17–19, 23–25] as well as in the documentation for the participating hospitals [26].

### SSI intervention

The SSI intervention needed each participating hospital to establish a multidisciplinary group (project team) responsible for optimizing the three main components (intervention bundle) of preoperative management, adapted from the WHO guidelines:

1. Preoperative hair removal/shortening;
2. Preoperative skin disinfection [27]; and
3. Perioperative antibiotic prophylaxis, including optimal timing and repetition in case of prolonged duration and weight-adapted dosing [24]. The main intervention direct observations are listed in Table 1.

**Table 1:** Three main components of the SSI intervention

Patient data (weight; type of surgery)	
1. Adequate preoperative hair removal at the surgical site	Hair removal ONLY if indicated for surgical reasons
	Only through hair clipping or chemical depilatory. *No shaving/razors (inadequate)*
	Timing: on the day of surgery, within 4 h before incision (chemical depilation may be conducted already on the day before)
2. Adequate preoperative skin disinfection of the operation site	Three applications, while respecting exposure times after each application as per the manufacturer’s recommendations
	Carried out (or supervised) by a defined trained staff member
	Use of alcohol-based chlorhexidine, octenidine, or PVP-iodine (except for mucosa and wounds, where alcohol-based disinfectants must not be used)
3. Adequate administration of antibiotics	Written guideline for adherence to correct time window as per recommendations [11]
	Intraoperative repeat dose as per recommendations (usually within 4 h for first/second-generation cephalosporins)
	Weight adaptation, with dose increase at 80 kg cutoff

The implementation process included activities related to “leadership”, “standards”, and “training”. The project team led the use of structural and process quality parameters as indicators to determine successful implementation, including the structured introduction of local guidelines on the internationally accepted standards for preventing SSIs. Hospitals were asked to provide data on monitoring compliance with the national SSI intervention bundle for at least ten procedures every quarter for at least three years. A detailed description of the intervention is provided in Additional file 1: Supplementary Information.

### Primary outcome and data variables

The primary outcome was the SSI incidence over three years (calculated from when the intervention started at each hospital) compared to three years preintervention for cardiac, colon, and hip/knee replacement surgery. These are standard procedures (except for cardiac surgery being performed only in larger centers) and can be considered representative indicators of SSI in Swiss acute care hospitals.

The variable SSI included any superficial or deep incisional infection and/or organ-space infection at 30 days and/or one year. Covariables included sex, age, body mass index (BMI), American Society of Anesthesiologists (ASA) score, wound contamination class: clean (class I), clean-contaminated (class II) or contaminated (class III), elective vs emergency procedure, and procedure duration exceeding standard time (T score). Wound contamination class IV was excluded since this category involves preexisting infection. SSI cases were classified according to Centers for Disease Control and Prevention (CDC) definitions [28]. The process of classifying SSIs under the national surveillance system is described in Additional file 1: Supplementary Information.

To analyze the influence of preoperative comorbidity, patients were grouped into low (1 or 2) and high ASA scores (3–5). Regarding bed size, hospitals were grouped into < 200 beds, 200–500 beds, and > 500 beds.

### Statistical analysis

To investigate differences in baseline characteristics between the pre- and postintervention periods, we used the *χ*^2^ or Wilcoxon tests for categorical and continuous data, respectively.

Poisson generalized linear mixed models were fitted to quarterly procedure-specific SSI rates and adjusted for hospitals and procedure types (random effects). Denominator data were the number of procedures per quarter per hospital. SSI rates for the three years before the intervention vs the first three years of the intervention were compared, taking into account a 3-month wash-out phase after the start of the intervention.

In addition to the Poisson model, an individual patient model (logistic mixed-effects regression) with SSI as the dependent variable was built. The main exposure was surgery after the intervention, and the model was adjusted for hospitals and procedure types (random effects) and the patient-related covariables: age, sex, BMI, ASA score, contamination class, elective and T score (fixed effects).

The significance level was set at *P* < 0.05 using a 2-sided test. All statistics were performed in R. Graphical displays of pre- and postintervention SSI rates followed an interrupted time series approach.

### Ethics approval

SSI surveillance by Swissnoso is mandated by Swiss healthcare policies and is considered a quality improvement project. All patients were informed about their automatic inclusion in SSI surveillance on admission and allowed to opt out. Summary results of the SSI incidences are published yearly [17, 25]. The Bernese Cantonal Ethics Committee (KEK) approved risk factor analyses within the SSI surveillance database (KEK #2019–00294).

### Reporting

The study follows the SQUIRE reporting guidelines for quality improvement studies [29].

## Results

The study included N = 10 151 patients (n = 5 489 preintervention and n = 4 662 postintervention). The patient age was similar during the pre- and postintervention phases, with a median of 69.6 (IQR 60.9 to 76.8) years vs. 69.5 (IQR 60.4 to 76.8) years, respectively (*P* = 0.55). The proportion of females was similar in both phases, accounting for 44.8% preintervention and 46.1% postintervention (*P* = 0.23). Knee/hip implantations were the most frequent procedure, followed by cardiac and colon surgery. The proportions of procedures differed significantly between preintervention and postintervention (*P* < 0.001), with lower numbers of colon surgeries and higher numbers of cardiac surgeries observed during preintervention than postintervention (25% vs. 29% and 31% vs. 27%, respectively). In contrast, the numbers of hip/knee surgeries were similar between both periods. There was a high proportion of elective surgery both pre- and postintervention, which was significantly higher preintervention (85.6%) than post-intervention (83.1%, *P* < 0.001). The median time of antimicrobial administration relative to incision was significantly longer preintervention (43 min; IQR, − 55 to − 31) than postintervention (35 min; − 49 to − 23, *P* < 0.001). The proportion of surgeries exceeding the standard time was similar in both phases. Of note, the number of procedures included varied across the different hospitals, reflecting the different hospital sizes (e.g., hospitals 2 and 6 were smaller centers). The patient baseline characteristics pre- vs postintervention included are shown in Table 2 (baseline characteristics for specific procedures are shown in eTable 1, Additional file 2).

**Table 2:** Comparison of overall patient baseline characteristics for the pre- vs postintervention phases, with *P* values

Baseline parameters	Preintervention	Postintervention	*P* value
N = 10 151	5489	4662	
Age (median [IQR])	69.60 [60.87 to 76.84]	69.46 [60.37 to 76.83]	0.55
Sex = female (%)	2461 (44.8)	2147 (46.1)	0.23
ASA score (%)			0.02
1 or 2	2493 (45.4)	2042 (43.8)	
3–5	2987 (54.4)	2601 (55.8)	
Missing information	9 (0.2)	19 (0.4)	
BMI kg/m2 (median, IQR)	26.9 (24.1, 30.5)	26.7 (23.7, 30.0)	0.001
Wound contamination class (%)			< 0.001
I (clean)	4112 (74.9)	3272 (70.2)	
II (clean-contaminated)	1166 (21.2)	1203 (25.8)	
III (contaminated)	211 (3.8)	187 (4.0)	
Procedure			< 0.001
Colon surgery	1344 (24.5)	1358 (29.1)	
Cardiac surgery	1682 (30.6)	1245 (26.7)	
Knee/hip implantation	2463 (44.9)	2059 (44.2)	
Elective surgery = yes (%)	4696 (85.6)	3873 (83.1)	0.001
Antibiotic administration in relation to incision (median [IQR])	− 43 [− 55 to − 31]	− 35 [− 49 to − 23]	< 0.001
Exceeding T score^a^ = yes (%)	1468 (26.7)	1154 (24.8)	0.02
Year (median [IQR])	2015 [2014 to 2016]	2018.00 [2017 to 2018]	< 0.001
Hospital (%)			< 0.001
1	794 (14.5)	841 (18.0)	
2	158 (2.9)	127 (2.7)	
3	873 (15.9)	846 (18.1)	
4	586 (10.7)	679 (14.6)	
5	1740 (31.7)	773 (16.6)	
6	31 (0.6)	60 (1.3)	
7	493 (9.0)	578 (12.4)	
8	814 (14.8)	758 (16.3)	

^a^T-score: number (%) of procedures where the duration exceeded the 75th percentile of the standard operation duration

In the 3-year preintervention phase, 309 SSIs (5.6%) were observed, and 226 (4.8%) were observed within the three years postintervention (*P* = 0.09). Interyear and intrayear variations in quarterly SSI rates related to intervention start were observed for all procedures, with the most prominent variations seen in colon and cardiac surgery.

**Fig. 1:**
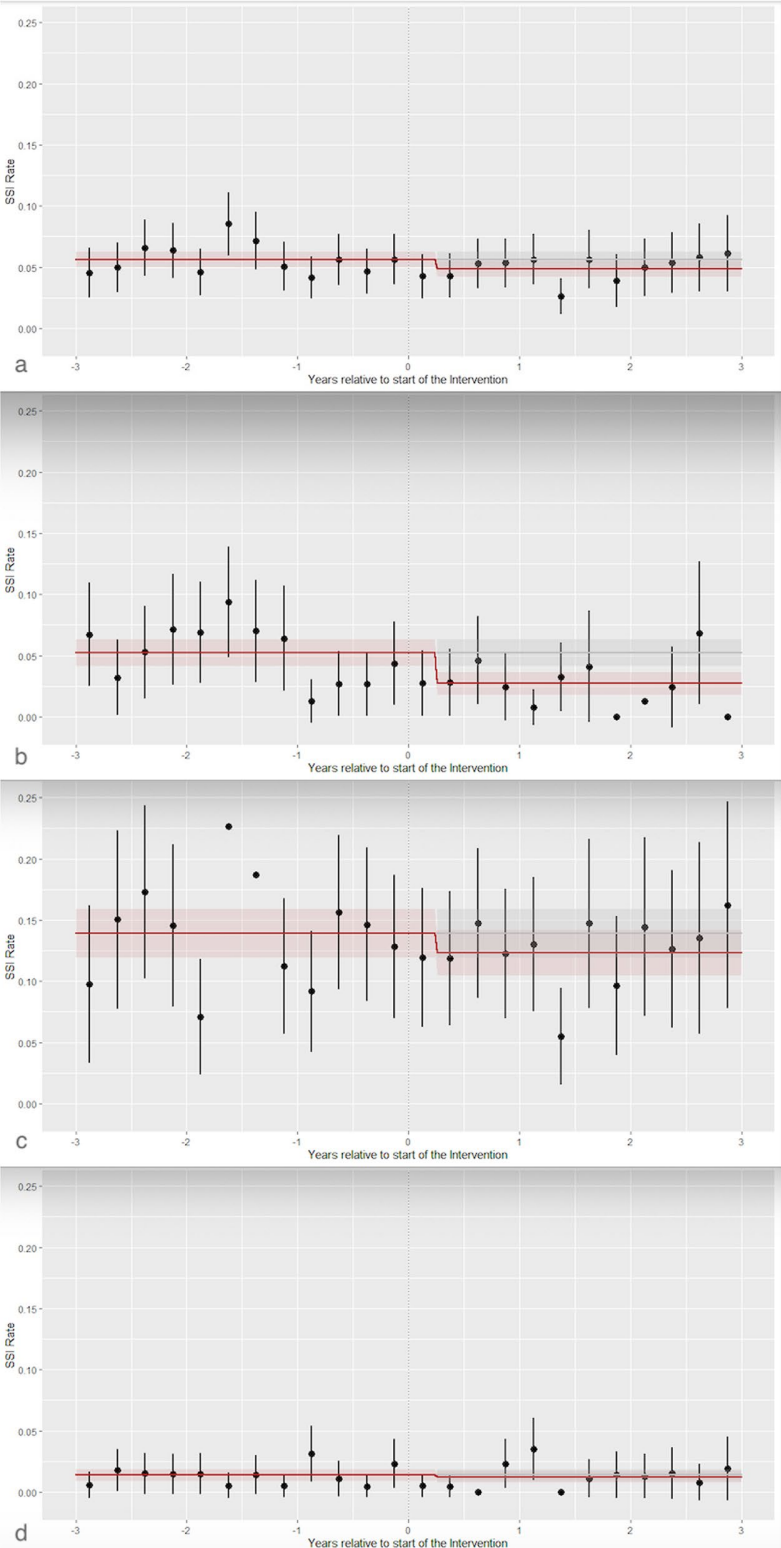
Quarterly SSI rates over the study period with pointwise 95% CIs (black) with fitted interrupted Poisson generalized linear model (red line) with interruption at time 0 (start of the intervention) and a 3-month wash-out phase. SSIs for **a** all procedures; **b** cardiac surgery; **c** colon surgery; and **d** knee/hip replacement. *SSI, surgical site infection*

The intervention was associated with a statistically significant, lower adjusted SSI incidence rate ratio (aIRR) of 0.81 (95% CI, 0.68 to 0.96, *P* = 0.02). Regarding specific types of procedures, cardiac surgery was associated with the lowest intervention aIRR of 0.48 in cardiac surgery patients (95% CI, 0.32 to 0.72, *P* < 0.001). The crude SSI incidence before/after the intervention for cardiac surgery hospitals is shown in eTable 1 in the Additional file 2 section. In colon surgery, with an aIRR of 0.93 (95% CI, 0.75 to 1.15, *P* = 0.49), and in hip/knee replacement, an aIRR of 0.88 (95% CI, 0.52 to 1.48, *P* = 0.63), there was no significant association between the intervention and SSI. Quarterly SSI rates with 95% confidence intervals overall are shown in Fig. 1a and for specific procedures in Fig. 1b-d.

In the individual patient fixed effects model, the intervention was significantly associated with a decreased adjusted SSI OR (0.70, 95% CI, 0.56 to 0.88, *P* = 0.002) (Table 3).

**Table 3:** Additional patient-based model with odds ratios adjusted (aORs) for additional covariables of age, sex, ASA score, contamination class, elective and T score (fixed effects), and body mass index (BMI)

	aOR	95% CI	*P* value
Postintervention (ref = preintervention)	0.70	0.56 to 0.88	0.003
Age (per year increase)	1.00	0.99 to 1.01	0.78
Sex = female, (ref = male)	0.95	0.76 to 1.19	0.65
ASA score 3–5 (ref = ASA 1 and 2)	2.10	1.56 to 2.82	< 0.001
Wound contamination class II (clean-contaminated) Ref = class 1 (clean)	4.14	1.36 to 12.57	0.01
Wound contamination class III (contaminated) Ref = class 1 (clean)	3.76	1.32 to 10.75	0.01
Elective surgery (Ref = emergency surgery)	0.67	0.52 to 0.86	0.002
Exceeding T score = yes^a^ (Ref = no)	1.20	0.94 to 1.53	0.136
BMI per kg/m2 increase	1.03	1.01 to 1.05	0.003

^a^T-score: number (%) of procedures where the duration exceeded the 75th percentile of the operation duration

### Monitoring compliance with the national SSI intervention bundle

**Fig. 2:**
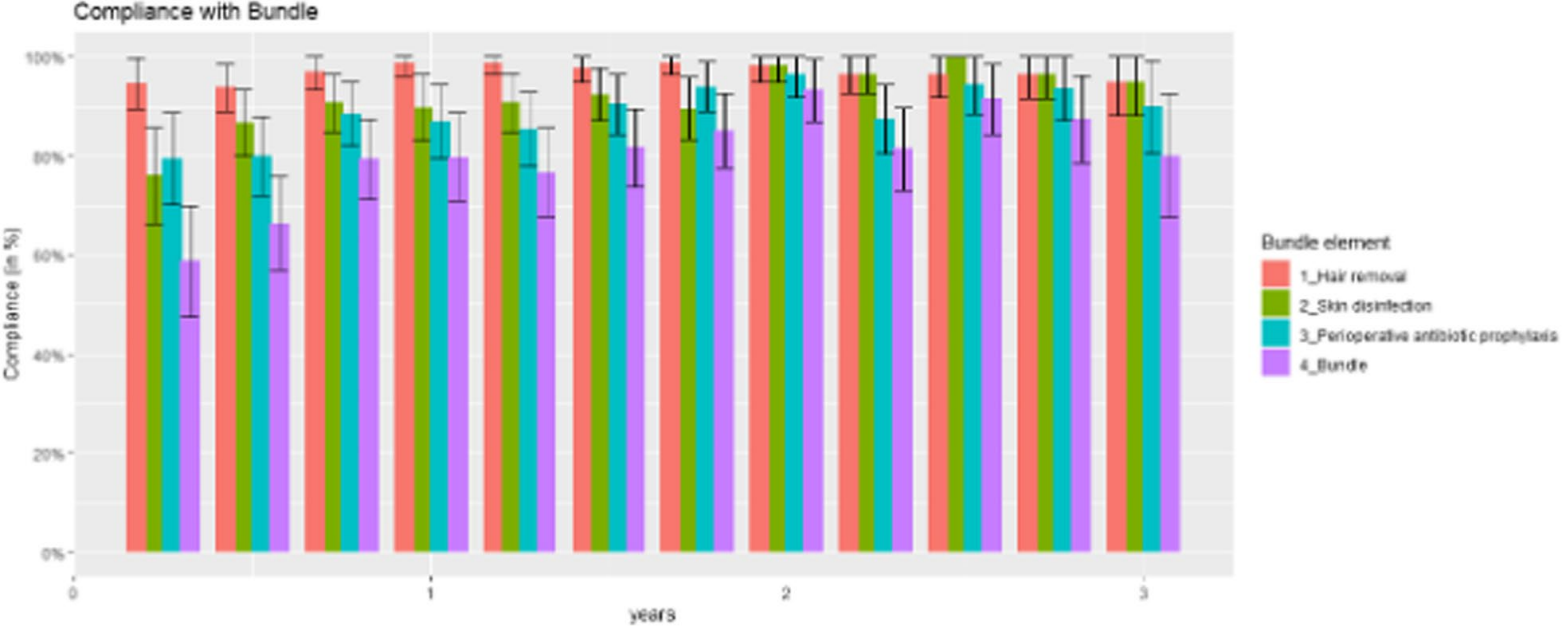
Intervention bundle adherence by quarter, from Q1 year 1 to Q4 year 3, after local implementation of the intervention (N = 916 observations). Compliance with each element of preoperative preparation (red = correct hair removal, green = correct skin disinfection, turquoise = correct administration of antibiotic prophylaxis) is shown as a rate with 95% CIs per quarter. In purple, the achievement of the entire bundle (simultaneous fulfilment of all three elements) is shown

Compliance data from all participating hospitals included 916 observations (chosen by the individual hospitals according to their local priorities) for three years after introducing the intervention at the hospitals. Overall bundle adherence increased significantly from 59% (95% CI, 48% to 70%) at baseline to 80% (95% CI, 68% to 94%) at the end of year 3 (*P* = 0.03) since the start of the intervention. Errors occurred due to disinfectants without remanence and/or applying the disinfectant and/or antimicrobial prophylaxis outside the recommended times. The best compliance was achieved with hair removal in Q1 of the second year since intervention (97%). Compliance data are shown in Fig. 2 and eTable 2, Additional file 3.

## Discussion

### Principal findings

The adjusted results show that adding targeted SSI intervention bundles to the surveillance system was associated with a nearly 20% statistically significant lower overall SSI IRR. This suggests that implementing SSI intervention modules on a national level may provide additional value to further decrease the rates of SSI. To our knowledge, no comparable published work has implemented a prevention bundle to compare SSI rates pre- and postintervention for different types of surgery.

The strongest association between intervention and SSI was detected in cardiac surgery, with an aIRR of ~ 0.5. In contrast, the association was less prominent and nonsignificant for the other types of surgery. This finding seems plausible since the intervention might have been best adopted in the context of these complex (cardiac) procedures. In addition, a higher baseline of infection rates in cardiac surgery (3.3%) provided a greater potential for optimizing presurgical management and reducing incidence rates compared to generally lower hip (1.2%) and knee (0.9%) SSI throughout Swiss hospitals [17]. Our results corroborate findings from Vos et al., who, in their systematic literature review, identified optimized perioperative prophylaxis by prolonged use of a first-generation cephalosporin [30] as an essential measure for preventing deep sternal wound infections in cardiac surgery. Likewise, our intervention included intraoperative repeat doses per recommendations (usually within 4 h for first/second-generation cephalosporins).

A reduction in SSI rates was also shown by Schweizer et al., who reported a minor decrease in complex *S. aureus* infections for cardiac surgery and a slightly more pronounced decrease for hip or knee surgery [19]. Their intervention focused on preoperative decolonization and perioperative prophylaxis according to an individual’s methicillin-sensitive (MSSA) and methicillin-resistant *S. aureus* (MRSA) status [18]. For hip and knee surgery, we detected a lower postintervention SSI of ~ 10%, with large confidence intervals that were not statistically significant. With baseline infection rates of only approximately 1% in clean orthopedic surgery, even a large sample size would have been unlikely to detect a significant effect. In addition, the improvement from baseline was likely not sufficiently high to demonstrate a statistically significant effect. Vicentini et al. [20] in their systematic review, identified *S. aureus* detection and decolonization as an effective mechanism for reducing hip replacement SSI and underlined the importance of including appropriate hair removal and adequate preoperative antibiotic prophylaxis. 

Our results showed a 7% lower SSI aIRR for colon surgery. An explanation for this could be that other variables, such as preoperative colon decolonization, might play a greater role in infection prevention in these procedure types [31, 32]. Jurt et al. [33] in their small single-center study in Switzerland, employed a standardized intraoperative care bundle (timing and repeat dose of antibiotic prophylaxis, among others) for colon surgery and detected no association with lower SSI rates. The authors assumed there might have been insufficient compliance in the most critical steps of the intervention (related to wound protection and closure, among others), highlighting the complexities in a referral center and the fact that SSI rates were comparable with national figures previously described [33]. Oral antibiotic prophylaxis prior to surgery was not part of our bundle. More than in the past, this will need to be considered for future gastrointestinal decolonization bundles. Keenan et al. [34] in their retrospective study on SSI rates following the implementation of a preventive SSI bundle for colorectal surgery in the United States, detected a substantial reduction in superficial but not in deep/organ-space SSIs. The systematic review of Pop-Vicas et al. [35] found that prevention bundles containing 11 or more elements showed the most significant SSI reduction but only included three randomized controlled trials, whereas high clinical/bundle heterogeneity and low quality were reported for most observational studies.

In all, the large patient numbers included in our study and reliance on mechanisms of an established national surveillance system strengthen the quality of our findings. Furthermore, we demonstrated high (or, in areas with low baseline rates, increasing) compliance with the different components of preoperative management (hair removal, skin disinfection, and perioperative antimicrobial prophylaxis) achieved by the intervention activities used through regular feedback and quality improvement activities.

### Internal and external validity

Our findings provide internal validity due to smaller (district) and more extensive (including tertiary care) hospitals being included, even though an overall moderate number of centers were included (and not all of them provided major procedures such as cardiac surgery). In addition, we estimate that external validity for similar healthcare settings outside the Swiss acute care hospitals is adequate for where SSI interventions are implemented in addition to functioning SSI surveillance systems.

### Clinical and research implications

A specific intervention program may, therefore, provide an additional benefit compared to surveillance alone. The results will be corroborated by more comprehensive implementation and enrollment of more hospitals to allow further evaluation of impact and to determine areas to be improved (considering adding additional bundle components and modifying/adding implementation/quality tools). Moreover, examining potential effects for a broader range of surgical procedures will help to further validate the benefit of SSI intervention.

Based on these results, the Swiss SSI intervention was further elaborated and offered to all Swiss acute care hospitals already performing SSI monitoring. A process monitoring app is now also available on mobile devices. This app allows for simplified and immediate entry of observed processes, and all results can be viewed immediately on a secure website for ongoing self-evaluation and benchmarking purposes. The intervention was expanded in 2023 by including preoperative *S. aureus* decolonization, preoperative gut decolonization and perioperative glycemic control [36].

While Switzerland is still transitioning from hospitals adding interventions to the national surveillance to measure the potential impact on SSI rates, future work will help explore the effects of the additional bundle elements across different hospital settings and the role of other types of surgical procedures. For example, mechanisms for a broad, automated collection of standardized process data facilitate an effective evaluation of the potential impact of interventions and should be explored in the future.

### Study limitations

Our study included a large number of patients and featured a pragmatic, multicenter design. One limitation of our study was the moderate number of participating hospitals, particularly for cardiac surgery (one hospital showed a particularly strong decrease in SSI), in contrast to the other types of surgery. This and differing proportions of elective procedures, albeit in both groups near 85%, may have introduced bias affecting the association between intervention and the aIRR for SSI rates. Another limitation is that the SSI surveillance program predefined variables and, therefore, did not include patient data on comorbidities, intraoperative data, or other potentially relevant details (e.g., whether bowel preparation had been performed) that would have allowed examination of further associations, a fact described previously [31].

This observational pre- and postintervention study did not include randomization or measure changes over time. Therefore, the association between bundle implementation and reduced SSI rates does not imply causality. Along these lines, the reduced SSI rates in the eight participating hospitals were not compared to those in nonparticipating hospitals. The effect might, therefore, not be solely attributable to the intervention. On the other hand, such a comparison would have likely been prone to bias due to hospitals starting the intervention at different times and potential spillover effects of prevention methods to nonparticipating hospitals. While confounding has been accounted for by adjusting rate ratios for relevant factors, changes in the case mix or in the referral pathways (less complex surgical procedures might have been transferred to the outpatient setting or smaller centers) might have introduced bias due to which important associations would have been missed.

Not all procedures were screened for compliance with the process parameters. There may have been a selection bias toward observing scheduled routine surgery and, therefore, a potential overestimation of bundle compliance. It might be the case that the intervention was adopted to a higher degree in complex procedures (such as cardiac surgery), but our study methodology did not include systematic measurement of bundle adherence for procedures (or their different levels of complexity); therefore, it did not allow determining the potential effect on SSI rates. Cardiac surgery has considerable complexity and may therefore rely more on the current bundle intervention than other procedures (potentially working better on clean vs. contaminated surgery). Additionally, higher baseline infection rates compared to hip/knee arthroplasty render it a procedure type with considerable potential to reduce SSI rates postintervention.

## Conclusions

The introduction of the SSI intervention bundle was associated with a statistically significant decrease in overall SSI cases. The strongest association was observed in cardiac surgery. The addition of a specific intervention program can provide added value compared to routine surveillance alone. Further prevention modules are necessary for colon and orthopedic surgery.

### Supplementary Information

**Additional file 1**. Supplementary Information.

**Additional file 2**. eTable 1.

**Additional file 3**. eTable 2.

## Data Availability

Anonymized data from the mandatory Swiss national surveillance of surgical site infections can be requested from the board at Swissnoso, the National Center for Infection prevention, in writing.

## Abbreviations

aIRR: Adjusted incidence rate ratio
aOR: Adjusted odds ratio
ASA: American society of anesthesiologists score
BMI: Body mass index
CDC: Centers for disease control and prevention
CI: Confidence interval
IQR: Interquartile range
MSSA/MRSA: Methicillin-sensitive/methicillin-resistant *Staphylococcus aureus*
SSI: Surgical site infection
