# Supplementary material for: Association between the introduction of a national targeted intervention program and the incidence of surgical site infections in Swiss acute care hospitals
Source: Antimicrob Resist Infect Control. 2023 Nov 24;12:134. doi: 10.1186/s13756-023-01336-7 (PMC10668371; doi:10.1186/s13756-023-01336-7)
Supplement: Supplementary file 1 — Additional file 1. Supplementary Information. [file 13756_2023_1336_MOESM1_ESM.docx]

**Additional file 1: Supplementary Information**

**Further information on materials and methods**

*Study Design and Data Sources*

**Description of SSI surveillance:**

The Swiss SSI surveillance system requires each participating hospital to collect data for all patients undergoing at least three different types of procedures during a selected period.^17^ Patients have the option to opt out. Surveillance includes data collection at discharge followed by rigorous postdischarge follow-up 30 days after the intervention with additional chart review in case of suspected infection.^17^ For implant surgery, a second follow-up takes place at one year. For each patient, at least five contact attempts are made over the phone before they are classified as “lost to follow-up”.

Data collection and recording in the national database is a standardized process combined with a separate audit process periodically conducted by Swissnoso staff to minimize bias between hospitals.^19^

*SSI Intervention*

**Detailed description of the intervention**

This included measuring overall bundle compliance and compliance with the three components based on observed process parameters. Data were submitted by all designated team members at the participating hospitals using a standardized monitoring tool. No maximum number of observations or specific sample size was set for the hospitals. As part of the quality tool, the above information (and individual observations) was submitted to the project managers quarterly, including a benchmark that allowed hospitals to compare their performance with other participating facilities. The feedback mechanism was aimed at improving overall quality.

**Leadership (translated excerpt of the participant handbook)** ^26^

**Preintervention:**

- The hospital management officially supports the intervention, including the required resources (letter).

- A project leader for the hospital is defined

- The project leader has attended a Swissnoso training session on the intervention module.

- A project group was formed, consisting of the project leader, two physicians (from the disciplines of surgery and anesthesia), one surgical nurse and one person from the hospital administration.

- The (surgical) discipline(s) to be observed have been determined

- Written guidelines for the individual elements are already in place

- Responsible site personnel have been/will be trained in implementing the guidelines; this includes training of the appropriate target groups in preoperative hair removal/shortening, skin disinfection, perioperative antibiotic prophylaxis, preoperative *Staphylococcus aureus* decolonization, preoperative bowel decolonization, and perioperative blood glucose monitoring.

**During the intervention**

- Regular communication with the Swissnoso project leader to clarify questions and discuss problems concerning the intervention implementation.

- Optimal participation in telephone conferences with the other participating hospitals (approx. every 6-10 weeks)

- Participation in workshops organized by Swissnoso

*Primary Outcome and Data Variables*

**SSI classification (primary outcome parameters)**

Surveillance staff reviewed all patient data, and a dedicated physician cross-checked those patients with suspected SSI. All supervising physicians – the majority were board-certified in infectious diseases – and infection control nurses had attended a training course on SSI surveillance. Data were electronically entered into a centralized database. The type of SSI – superficial incisional, deep incisional, or organ space – was recorded, as well as the pathogen (if available). Primary data were obtained from the patient charts and telephone interviews with patients. The data source for the variables was the Swissnoso SSI surveillance program.
