# Supplementary material for: Association between the introduction of a national targeted intervention program and the incidence of surgical site infections in Swiss acute care hospitals
Source: Antimicrob Resist Infect Control. 2023 Nov 24;12:134. doi: 10.1186/s13756-023-01336-7 (PMC10668371; doi:10.1186/s13756-023-01336-7)
Supplement: Supplementary file 2 — Additional file 2. eTable 1. [file 13756_2023_1336_MOESM2_ESM.docx]

**Additional file 2: eTable 1** Comparison of the baseline characteristics of the patients for the pre- and postintervention phases, with *P* values. Seventy-fifth percentile of the duration of operation (T score) for (a) cardiac, (b) hip and knee, and (c) colon surgery. NA: missing information; NA: not applicable; nonnorm: nonparametric test.

^a^T-score: number (%) of procedures where the duration exceeded the 75th percentile of the operation duration.

^b^ Cardiac procedure type:
CARD: Cardiac surgery excluding coronary artery bypass grafting, vascular surgery, transplantation, or permanent pacemaker implantation.

PCA: Coronary artery bypass grafting(s) involving venous transplant or peripheral artery (A. radialis)

PCAV: Coronary artery bypass grafting(s) involving A. mammaria interna or A. thoracica.

(a) Cardiac

|  | Preintervention | Postintervention | *P* value | Test |
| --- | --- | --- | --- | --- |
| n = 2 927 | 1 682 | 1 245 |  |  |
| Age (median [IQR]) | 69.16 [61.02 to 75.65] | 69.84 [60.62 to 76.06] | 0.63 | nonnorm |
| Sex = female (%) | 417 (24.8) | 325 (26.1) | 0.44 |  |
| ASA score (%) |  |  | 0.69 |  |
| 1 or 2 | 79 (4.7) | 62 (5.0) |  |  |
| 3-5 | 1 600 (95.1) | 1 179 (94.7) |  |  |
| NA | 3 (0.2) | 4 (0.3) |  |  |
| Wound contamination class (%) |  |  | 0.06 |  |
| I (clean) | 1 655 (98.4) | 1 233 (99.0) |  |  |
| II (clean-contaminated) | 11 (0.7) | 1 (0.1) |  |  |
| III (contaminated) | 16 (1.0) | 11 (0.9) |  |  |
| Hospital (%) |  |  | NA |  |
| 1 | 0 (0.0) | 0 (0.0) |  |  |
| 2 | 0 (0.0) | 0 (0.0) |  |  |
| 3 | 873 (51.9) | 846 (68.0) |  |  |
| 4 | 0 (0.0) | 0 (0.0) |  |  |
| 5 | 809 (48.1) | 399 (32.0) |  |  |
| 6 | 0 (0.0) | 0 (0.0) |  |  |
| 7 | 0 (0.0) | 0 (0.0) |  |  |
| 8 | 0 (0.0) | 0 (0.0) |  |  |
| Elective surgery = yes (%) | 1172 (69.7) | 763 (61.3) | <0.001 |  |
| Antibiotic administration in relation to incision (median [IQR]) | -43.00 [−56.00 to −30.00] | −30.00 [−43.00 to −20.00] | <0.001 | nonnorm |
| Exceeding T score^a^ = yes (%) | 298 (17.7) | 264 (21.2) | 0.02 |  |
| CARD^b^ | 924 (54.9) | 672 (54.0) | 0.64 |  |
| PCA^b^ | 857 (51.0) | 500 (40.2) | <0.001 |  |
| PCAV^b^ | 747 (44.4) | 646 (51.9) | <0.001 |  |
| Cardiac_implant: |  |  | <0.001 |  |
| 1 Cerclage (metal wires) | 885 (52.6) | 657 (52.8) |  |  |
| 2 Valve (mechanical or tissue) | 647 (38.5) | 369 (29.6) |  |  |
| 3 Patches to the cardiac wall | 14 (0.8) | 7 (0.6) |  |  |
| 4 other | 71 (4.2) | 104 (8.4) |  |  |
| Not applicable | 65 (3.9) | 108 (8.7) |  |  |

(b) Knee/hip

|  | Preintervention | Postintervention | *P* value | Test |
| --- | --- | --- | --- | --- |
| n = 4 522 | 2 463 | 2 059 |  |  |
| Age (median [IQR]) | 70.16 [61.97 to 77.39] | 69.98 [61.60 to 77.34] | 0.47 | nonnorm |
| Sex = female (%) | 1 364 (55.4) | 1 117 (54.2) | 0.47 |  |
| ASA score (%) |  |  | 0.08 |  |
| 1 or 2 | 1 630 (66.2) | 1 300 (63.1) |  |  |
| 3-5 | 828 (33.6) | 752 (36.5) |  |  |
| NA | 5 (0.2) | 7 (0.3) |  |  |
| Wound contamination class (%) |  |  | 0.004 |  |
| I (clean) | 2 457 (99.8) | 2 039 (99.0) |  |  |
| II (clean-contaminated) | 5 (0.2) | 19 (0.9) |  |  |
| III (contaminated) | 1 (0.0) | 1 (0.0) |  |  |
| Hospital (%) |  |  | NA |  |
| 1 | 520 (21.1) | 522 (25.4) |  |  |
| 2 | 139 (5.6) | 111 (5.4) |  |  |
| 3 | 0 (0.0) | 0 (0.0) |  |  |
| 4 | 586 (23.8) | 679 (33.0) |  |  |
| 5 | 585 (23.8) | 218 (10.6) |  |  |
| 6 | 0 (0.0) | 0 (0.0) |  |  |
| 7 | 31 (1.3) | 0 (0.0) |  |  |
| 8 | 602 (24.4) | 529 (25.7) |  |  |
| Elective surgery = yes (%) | 2 457 (99.8) | 2 058 (100.0) | 0.2 |  |
| Antibiotic administration in relation to incision (median [IQR]) | −40.00 [−50.00 to −31.00] | −33.00 [−45.00 to −20.00] | <0.001 | nonnorm |
| Exceeding T score^a^ = yes (%) | 529 (21.5) | 260 (2.6) | <0.001 |  |

(c) Colon

|  | Preintervention | Postintervention | *P* value | Test | |  |
| --- | --- | --- | --- | --- | --- | --- |
| N = 2 702 | 1 344 | 1 358 |  |  | |  |
| Age (median [IQR]) | 68.62 [57.59 to 77.14] | 68.14 [57.69 to 77.43] | 0.701 | nonnorm | |  |
| Sex = female (%) | 680 (50.6) | 705 (51.9) | 0.517 |  | |  |
| ASA score (%) |  |  | <0.001 |  | |  |
| 1 or 2 | 784 (58.3) | 680 (50.1) |  |  | |  |
| 3-5 | 559 (41.6) | 670 (49.3) |  |  | |  |
| NA | 1 (0.1) | 8 (0.6) |  |  | |  |
| Wound contamination class (%) |  |  | 0.27 |  | |  |
| II (clean-contaminated) | 1 150 (85.6) | 1 183 (87.1) |  |  | |  |
| III (contaminated) | 194 (14.4) | 175 (12.9) |  |  | |  |
| Hospital (%) |  |  | NA |  | |  |
| 1 | 274 (20.4) | 319 (23.5) |  |  | |  |
| 2 | 19 (1.4) | 16 (1.2) |  |  | |  |
| 3 | 0 (0.0) | 0 (0.0) |  |  | |  |
| 4 | 0 (0.0) | 0 (0.0) |  |  | |  |
| 5 | 346 (25.7) | 156 (11.5) |  |  | |  |
| 6 | 31 (2.3) | 60 (4.4) |  |  | |  |
| 7 | 462 (34.4) | 578 (42.6) |  |  | |  |
| 8 | 212 (15.8) | 229 (16.9) |  |  | |  |
| Elective surgery = yes (%) | 1 067 (79.4) | 1 052 (77.5) | 0.243 |  | |  |
| Antibiotic administration in relation to incision (median [IQR]) | −49.00 [−66.00 to −34.00] | −44.00 [−59.00 to −30.00] | <0.001 | nonnorm | |  |
| Exceeding T score^a^ = yes (%) | 641 (47.7) | 630 (46.4) | 0.523 | |  | |
