# Supplementary material for: Association between the introduction of a national targeted intervention program and the incidence of surgical site infections in Swiss acute care hospitals
Source: Antimicrob Resist Infect Control. 2023 Nov 24;12:134. doi: 10.1186/s13756-023-01336-7 (PMC10668371; doi:10.1186/s13756-023-01336-7)
Supplement: Supplementary file 3 — Additional file 3. eTable 2. [file 13756_2023_1336_MOESM3_ESM.docx]

**Additional file 3: eTable 2.** Aggregated audit data on adherence during the intervention period (N = 916 observations)

|  | Quarter | N obs | sum_compl | mean compl | Lower CI | Higher CI | Category intervention |
| --- | --- | --- | --- | --- | --- | --- | --- |
| 1 | 0.25 | 75 | 57 | 0.76 | 0.66 | 0.86 | 2_Skin disinfection |
| 2 | 0.5 | 98 | 85 | 0.87 | 0.80 | 0.93 | 2_Skin disinfection |
| 3 | 0.75 | 97 | 88 | 0.91 | 0.85 | 0.97 | 2_Skin disinfection |
| 4 | 1 | 79 | 71 | 0.90 | 0.83 | 0.97 | 2_Skin disinfection |
| 5 | 1.25 | 86 | 78 | 0.91 | 0.85 | 0.97 | 2_Skin disinfection |
| 6 | 1.5 | 93 | 86 | 0.92 | 0.87 | 0.98 | 2_Skin disinfection |
| 7 | 1.75 | 87 | 78 | 0.90 | 0.83 | 0.96 | 2_Skin disinfection |
| 8 | 2 | 60 | 59 | 0.98 | 0.95 | 1 | 2_Skin disinfection |
| 9 | 2.25 | 86 | 83 | 0.97 | 0.93 | 1 | 2_Skin disinfection |
| 10 | 2.5 | 59 | 59 | 1 | 1 | 1 | 2_Skin disinfection |
| 11 | 2.75 | 56 | 54 | 0.96 | 0.92 | 1 | 2_Skin disinfection |
| 12 | 3 | 40 | 38 | 0.95 | 0.88 | 1 | 2_Skin disinfection |
| 13 | 0.25 | 75 | 71 | 0.95 | 0.90 | 1.00 | 1_Hair removal |
| 14 | 0.5 | 98 | 92 | 0.94 | 0.89 | 0.99 | 1_Hair removal |
| 15 | 0.75 | 97 | 94 | 0.97 | 0.93 | 1 | 1_Hair removal |
| 16 | 1 | 79 | 78 | 0.99 | 0.96 | 1 | 1_Hair removal |
| 17 | 1.25 | 86 | 85 | 0.99 | 0.97 | 1 | 1_Hair removal |
| 18 | 1.5 | 93 | 91 | 0.98 | 0.95 | 1 | 1_Hair removal |
| 19 | 1.75 | 87 | 86 | 0.99 | 0.97 | 1 | 1_Hair removal |
| 20 | 2 | 60 | 59 | 0.98 | 0.95 | 1 | 1_Hair removal |
| 21 | 2.25 | 86 | 83 | 0.97 | 0.93 | 1 | 1_Hair removal |
| 22 | 2.5 | 59 | 57 | 0.97 | 0.92 | 1 | 1_Hair removal |
| 23 | 2.75 | 56 | 54 | 0.96 | 0.92 | 1 | 1_Hair removal |
| 24 | 3 | 40 | 38 | 0.95 | 0.88 | 1 | 1_Hair removal |
| 25 | 0.25 | 75 | 58 | 0.80 | 0.70 | 0.89 | 3_Perioperative antibiotic prophylaxis |
| 26 | 0.5 | 98 | 76 | 0.8 | 0.72 | 0.88 | 3_Perioperative antibiotic prophylaxis |
| 27 | 0.75 | 97 | 77 | 0.89 | 0.82 | 0.95 | 3_Perioperative antibiotic prophylaxis |
| 28 | 1 | 79 | 67 | 0.87 | 0.80 | 0.95 | 3_Perioperative antibiotic prophylaxis |
| 29 | 1.25 | 86 | 70 | 0.85 | 0.78 | 0.93 | 3_Perioperative antibiotic prophylaxis |
| 30 | 1.5 | 93 | 76 | 0.91 | 0.84 | 0.97 | 3_Perioperative antibiotic prophylaxis |
| 31 | 1.75 | 87 | 79 | 0.94 | 0.89 | 0.99 | 3_Perioperative antibiotic prophylaxis |
| 32 | 2 | 60 | 56 | 0.97 | 0.92 | 1 | 3_Perioperative antibiotic prophylaxis |
| 33 | 2.25 | 86 | 70 | 0.88 | 0.80 | 0.95 | 3_Perioperative antibiotic prophylaxis |
| 34 | 2.5 | 59 | 50 | 0.94 | 0.88 | 1 | 3_Perioperative antibiotic prophylaxis |
| 35 | 2.75 | 56 | 44 | 0.94 | 0.87 | 1 | 3_Perioperative antibiotic prophylaxis |
| 36 | 3 | 40 | 36 | 0.9 | 0.81 | 0.99 | 3_Perioperative antibiotic prophylaxis |
| 37 | 0.25 | 75 | 44 | 0.59 | 0.47 | 0.70 | 4_Bundle |
| 38 | 0.5 | 98 | 65 | 0.66 | 0.57 | 0.76 | 4_Bundle |
| 39 | 0.75 | 97 | 77 | 0.79 | 0.71 | 0.87 | 4_Bundle |
| 40 | 1 | 79 | 63 | 0.80 | 0.71 | 0.89 | 4_Bundle |
| 41 | 1.25 | 86 | 66 | 0.77 | 0.68 | 0.86 | 4_Bundle |
| 42 | 1.5 | 93 | 76 | 0.82 | 0.74 | 0.90 | 4_Bundle |
| 43 | 1.75 | 87 | 74 | 0.85 | 0.78 | 0.93 | 4_Bundle |
| 44 | 2 | 60 | 56 | 0.93 | 0.87 | 1.00 | 4_Bundle |
| 45 | 2.25 | 86 | 70 | 0.81 | 0.73 | 0.90 | 4_Bundle |
| 46 | 2.5 | 59 | 54 | 0.92 | 0.84 | 0.99 | 4_Bundle |
| 47 | 2.75 | 56 | 49 | 0.88 | 0.79 | 0.96 | 4_Bundle |
| 48 | 3 | 40 | 32 | 0.8 | 0.67 | 0.93 | 4_Bundle |

Compliance with the different elements of preoperative preparation (1 = skin disinfection, 2 = correct hair removal, 3 = administration of antibiotic prophylaxis, and 4 = overall bundle compliance) is shown as quarterly number of observations and compliance detected, mean rates with 95% CIs per quarter between the first quarter (Q1), 2017 and Q1, 2019.
